# Fermented bread, a promising additive to pig feed to improve intestinal health—an *in vitro* study

**DOI:** 10.7717/peerj.21397

**Published:** 2026-06-09

**Authors:** Messan Kokouvi Djakpa, Marie-Line Daumer, Pascal Peu, Frédérique Mayeur-Nickel, Sandra Wiart-Letort, Frédéric Dessauge, Myriam M.L. Grundy

**Affiliations:** 1 PEGASE, Institut National de la Recherche pour l’Agriculture, l’Alimentation et l’Environnement (INRAE), Saint-Gilles, France; 2 OPAALE, Institut National de la Recherche pour l’Agriculture, l’Alimentation et l’Environnement (INRAE), Rennes, France

**Keywords:** Fermentation, Dietary fibres, Volatile fatty acids, *In vitro* models, Intestinal barrier function, Pig, IPEC-J2, Digestion, Bread, Butyric acid

## Abstract

**Background:**

Weaning stress in piglets leads to digestive disorders and compromised intestinal barrier function. Dietary fibres and bioactive compounds like volatile fatty acids represent promising alternatives to antibiotics. This study investigated the potential of fermented bread as a sustainable feed additive to improve intestinal health in pigs through *in vitro* digestion and cell culture models.

**Methods:**

White bread (WB) and wholemeal bread (MB) were fermented anaerobically for 8 days at 35 °C. Bread samples and their fermented products (fermented white bread (FWB) and fermented wholemeal bread (FMB)) were analysed for their nutritional composition (protein, dietary fibres and volatile fatty acids), subjected to *in vitro* oro-gastrointestinal digestion using the INFOGEST protocol, and tested on porcine jejunal epithelial cells (IPEC-J2) for barrier function assessment. The cell viability and integrity of the IPEC-J2 were analysed by transepithelial electrical resistance, MTS assay, and immunohistochemistry for tight junction proteins (zonula occludens-1, ZO-1). Additionally, monitoring of the diffusion of FITC-dextran (FD4) and lucifer yellow (LY) through the cell monolayer was performed.

**Results:**

Overall, fermentation significantly altered the nutritional profile of the breads, increasing the volatile fatty acids content, particularly lactic and butyric acid (*e.g.*; in FMB 29.8 and 10.6 mg/g of total dry mass, respectively). *In vitro* digestion showed 93% mass loss when enzymes were present, indicating high digestibility. Cell culture experiments revealed that, when exposed to the digesta, the IPEC-J2 maintained their viability (> 90%) and barrier function, with only MB showing significant increases in LY permeability (*P* < 0.05). Fermented bread represents a promising sustainable feed additive that maintains intestinal barrier integrity while providing beneficial volatile fatty acids, particularly lactic acid and butyric acid, suggesting its potential as an antibiotic alternative in pig nutrition.

## Introduction

Weaning is a critical transitional period in pig production, characterised by multiple physiological and environmental stress factors that compromise digestive health and performance (Tang et al., 2022). These digestive disturbances have numerous physiological consequences, including compromised intestinal barrier function, and impaired growth, reduced feed intake and poor health, which in some cases can lead to death. This challenging period has traditionally prompted prophylactic antibiotic use, raising concerns about antimicrobial resistance development (Pluske, Turpin & Kim, 2018). The search for sustainable alternatives to antibiotics has led to increased interest in dietary fibres and bioactive compounds, particularly volatile fatty acids, as promising solutions for avoiding the use of antibiotics. Indeed, dietary fibres can be a central component of the piglet’s ration because they can promote intestinal health notably by modulating gut maturation and the microbiota (Bach Knudsen, Hedemann & Lærke, 2012). However, excessive dietary fibre content may compromise nutrient digestibility, particularly during the critical weaning period, since they are not degraded by the piglet’s enzymes in the upper digestive tract (Pluske, 2016). Despite this limitation, dietary fibres have numerous benefits for intestinal health in both the small intestine (*e.g.*, secretion of mucus, stimulation of epithelial cells, and facilitation of transit) and the colon (*e.g.*, microbiota enrichment and production of short-chain fatty acids through fermentation) (Jha & Berrocoso, 2015; Li et al., 2021).

Contemporary livestock production faces mounting pressure to adopt sustainable practices while maintaining productivity. In this context, food co-products can represent attractive solutions that address both waste management and nutritional requirements (Albizzati, Tonini & Astrup, 2021). In the bread-making industry, unsold bread is largely used in the production of bioenergy *via* methanisation (Cox et al., 2022). Recent French regulations and European Union directives prioritise food waste hierarchy management, emphasising feed applications over energy recovery (Code de l’environnement, 2020; Albizzati, Tonini & Astrup, 2021). Because of its high carbohydrate content, bread is an ideal substrate for fermentation processes that generate bioactive compounds including oligosaccharides and volatile fatty acids such as butyric acid (Dymchenko, Geršl & Gregor, 2023). Fermented bread could therefore be an alternative to synthetic butyric acid.

Both the products of fermentation (volatile fatty acids) and the remaining, unfermented dietary fibres could have a positive impact on intestinal health. Butyric acid, naturally produced by the intestinal microbiota, has been demonstrated to promote intestinal health and have anti-inflammatory properties in pig fed diets enriched in dietary fibres (Bach Knudsen et al., 2018). Indeed, previous studies using porcine jejunal epithelial cells (IPEC-J2) confirmed that butyric acid strengthened the integrity of the epithelial cell monolayer (Yan & Ajuwon, 2017; Kovanda et al., 2025; Wang et al., 2025). However, these studies used purified butyric acid, so the effect of more complex raw materials or ingredients (such as fermented bread) on intestinal health remains to be assessed. Furthermore, the importance of gastrointestinal digestion in generating physiologically relevant food matrices and accounting for molecular transformations during digestion remains underexplored. Oligosaccharides, like larger cell wall polysaccharides, may beneficially impact pig intestinal health through immune system modulation, enterocyte stimulation, and microbiota substrate provision (Liu et al., 2023).

It was with this in mind that this work was initiated, with the aim of studying the impact of fermented bread on the intestinal health of pigs using *in vitro* methods. The hypothesis of this study was that the dietary fibres and volatile fatty acids contained in the fermented breads had a positive impact on the intestinal barrier function. To test this hypothesis, first, a physicochemical characterisation was carried out on breads before and after fermentation to evaluate the level of degradation of the breads (dietary fibre but also starch and proteins). It was expected that this degradation would differ between white and wholemeal breads. Then, the breads and fermented products were digested *in vitro* using the INFOGEST protocol to simulate digestion in the upper digestive tract of pigs as previously performed (Grundy et al., 2022; Grondin et al., 2025). Finally, the obtained digesta were used as substrates on porcine jejunum epithelial cells (IPEC-J2) to evaluate their impact on the intestinal barrier function.

## Material and Methods

### Chemicals

Salivary amylase (A0521, 184 U/mg of solid), porcine pepsin (P6887, 2,326 U/mg of solid), porcine pancreatin (P7545, 3.8 U/mg of solid based on trypsin activity), porcine bile extract (B3883), and fluorescein isothiocyanate–dextran of 4 kDa (FD4) were purchased from Merck (Saint Quentin Fallavier, France). The porcine jejunal intestinal cell line, IPEC-J2, was obtained from DSMZ (ACC-701, Braunschweig, Germany). MTS 3-(4,5-dimethylthiazol-2-yl)-5-(3-carboxymethoxyphenyl)-2-(4-sulfophenyl)-2H-tetrazolium, internal salt reagent, was purchased from Promega (Charbonnières-les-Bains, France). Lucifer yellow (LY) and zonula occludens-1 (ZO-1) monoclonal antibody (mouse) Alexa FluorTM 594 were obtained from Thermo Fisher Scientific (Illkirch-Graffenstaden, France). All other chemicals, solvents and reagents were obtained from either Merck (Saint Quentin Fallavier, France) or Thermo Fisher Scientific (Illkirch-Graffenstaden, France).

### Bread fermentation

The breads were purchased in a local supermarket. A total of six loaves (three replicates for each type of bread) were included in this study: three white breads (WB) or French baguettes, and three wholemeal breads (MB) (Fig. S1). After a storage period of one week at room temperature, as would be expected to occur in a household, each bread was fermented under anaerobic conditions in water to obtain the fermented products (three fermented white bread, (FWB) and three fermented wholemeal bread (FMB)). Bread fermentation experiments were performed under non-sterile conditions in order to evaluate the spontaneous fermentation potential of bread waste. No external microbial inoculum was added and the fermentation relied on the endogenous microbiota naturally present in the bread matrix, originating from raw materials and the baking process. This approach was intentionally chosen to better reflect conditions that could realistically be implemented at industrial scale and/or at the farm, where sterilisation of large quantities of bread waste would not be used. Bread substrate and water were therefore used without prior sterilisation. However, all glassware and containers were thoroughly cleaned prior to the experiments to limit external contamination. Fermentations were conducted in closed jars in order to restrict oxygen transfer. The headspace was minimised and the containers remained sealed throughout the incubation. Under these conditions, oxygen was rapidly consumed by microbial activity, leading to the establishment of anaerobic conditions suitable for fermentation. Operational parameters were optimised with an experimental design. Thus, an aliquot of bread (250 g) was placed in a closed 2 L jar, containing 1 L of tap water, and kept under agitation (120 rpm; incubator IKA, KS 4000 i control, Staufen, Germany) at 35 °C during 8 days (see Fig. S1B).

### Nutritional analyses of breads and fermented products

The protein content of the breads before (solid form as showed in Fig. S1A) and after fermentation (liquid with particles see Fig. S1C) was measured with a LECO analyser using the Dumas method (LECO FP82, Villepinte, France; with a nitrogen conversion factor of 5.4). The Association of Official Agricultural Chemists (AOAC) method 2014.10 was used to obtain the starch content of WB and MB. Total dietary fibre content was estimated by measuring the soluble and insoluble dietary fibres fractions (method AOAC 991.43) of the two bread types, before and after fermentation. Standardised methods for dry matter (method AOAC 935.29) and ash (method AOAC 942.05) were also used. High pressure liquid chromatography (Dionex Ultimate 3000, Thermo Fisher Scientific, Waltham, MA, USA) on Supelcogel H column (Supelco) coupled with UV spectroscopy and an evaporation light scattering detector was used to determine the volatile fatty acids content of the samples. The sugars were quantified with the same column and equipment but with a refractometer as detector. All measurements were conducted in triplicate (*n* = 9 for each type of sample).

The neutral sugar composition (non-starch polysaccharides) of the breads before and after fermentation was determined by gas chromatography (Englyst & Cummings, 1988). Briefly, the alditol acetates were obtained by removal of the starch and protein, hydrolysis of the precipitated polysaccharides followed by a reduction and acetylation of their constitutive monosaccharides. The identification and quantification of the sugars was done using standards (L-rhamnose, D-fucose, L-arabinose, D-xylose, D-mannose, D-galactose and D-glucose) with inositol as internal standard. A GC-FID GC203 (Shimadzu, Marne la Vallée, France) equipped with a DB225-MS capillary column (30 m × 0.32 mm i.d. coated with, 0.25 µm film thickness; Thermo Fisher Scientific, Waltham, MA, USA) was used for the analysis. The volume of injection was 1 µL, split mode (ratio 1:50), with an injection temperature of 220 °C. The carrier gas was hydrogen (45 cm/s), the flow rate was one mL/min, and the oven temperature was set at 210 °C. Each analysis was performed in triplicate (*n* = 9 for each type of bread and fermented products).

### *In vitro* oro-gastrointestinal digestion

In order to evaluate how the breads, before and after fermentation, were degraded during their journey through the gastrointestinal tract, and to identify the proteins and peptides dispersed into the aqueous phase, an oro-gastrointestinal *in vitro* static digestion model was used (Brodkorb et al., 2019; Grundy et al., 2022). Each bread and fermented product were incubated with digestive simulated fluids (oral, gastric and then intestinal phases) as described in details elsewhere (Brodkorb et al., 2019). The incubation temperature was set at 39 °C to match the pig digestive conditions and the quantity of bread or fermented products added for the digestion was 5 g (five mL for the fermented products) as recommended by Brodkorb et al. (2019). For each bread and fermented product, two set of incubation was performed, either without (samples B, blank, protein solubilised) or with (samples T, test, hydrolysed protein) enzymes: salivary amylase, pepsin, and pancreatin for the oral, gastric and intestinal phases, respectively. The enzymes activity was stopped, either after the oral, gastric or intestinal phase, by placing the samples on ice and increasing the pH to 9. The samples were then centrifuged (4,000 g at 4 °C for 15 min) and the supernatant (stored at −20 °C until further analysis, notably for the IPEC-J2 experiments) and the pellet (dried at 60 °C overnight) collected. Each digestion was performed in triplicate (*n* = 9 for each type of bread and fermented products).

### Protein and peptide profile of breads, fermented products and the digesta

The proteins and peptides present in the supernatant following incubation (without or with enzymes) of the breads and fermented products were identified by Sodium Dodecyl Sulphate-Polyacrylamide Gel Electrophoresis (SDS-PAGE). The details of the method can be found elsewhere (Grundy et al., 2022).

### Cell culture and viability assay

The IPEC-J2 were grown in Dulbecco’s Modified Eagle Medium/Ham’s F-12 supplemented with 10% porcine serum and 1% penicillin-streptomycin as previously described (Perruchot et al., 2025). The passage used was between 3 and 6. Once the cells reached 80% confluence, they were seeded (1 × 10^5^ cells per cm^2^) onto 12-well plates with transwell polyester membrane inserts (0.4 µm pore size, 1.1 cm^2^ surface area). The culture medium contained DMEM/F12 medium, 5% PS, 1% penicillin-streptomycin, 1% insulin/transferrin/selenium, and 10 µg/mL epithelial growth factor. The cells were left to grow for 14 days under standard conditions (95% humidity, 37 °C and 5% CO_2_). The medium was changed every two to three days and the transepithelial electrical resistance measured on day 4, 7, 11 and 14 with an Epithelial Voltohmmeter (EVOM3, Friedberg, Germany). Cell viability was assessed for each type of bread and fermented products (*n* = 3), for both B (without enzymes) and T (with enzymes) samples using the MTS protocol. The digesta used for this viability assay, was recovered at the end of the intestinal phase, and detoxified (dilution 1:10 and 1:20 after heating at 100 °C for 5 min) as determined by Perruchot et al. (2025).

### Evaluation of the impact of bread and fermented product digesta on intestinal permeability

The MTS assay permitted to identify detoxification conditions where the digesta did not affect the viability of the IPEC-J2. Following these preliminary analyses, the supernatants detoxified (diluted at 1:10 and heated) were applied to the IPEC-J2 and the passage of FD4 (MW = 4,000 Da) and LY (MW = 522 Da) across the monolayers monitored (*n* = 3, one pool of digesta for each bread and fermented product) as previously described (Perruchot et al., 2025; Grondin et al., 2025).

The tight junction protein ZO-1 was observed by immunochemistry on the IPEC-J2 after exposition for 2 h to either cell media or detoxified digesta (bread and fermented products samples B and T, at the end of intestinal phase). The details of the protocol can be found in Perruchot et al. (2025) and Grondin et al. (2025). A Zeiss Apotome fluorescence microscope (40X objective) and the ImageJ software was used to obtain the images. These experiments were repeated in triplicate several weeks apart. For each replicate, two wells were used per condition, taking eight images per well (*n* = 6 for each type of bread and fermented product, with *n* = 1 for one representative image).

### Calculation and statistical analyses

R studio version 4.1.2 was used to analyse the data (expressed as means of replicates). The significance level was set at *P* < 0.05 (two tailed). Data were checked for normal distribution using Shapiro–Wilk test and homogeneity of variance with Levene test. Two-way analysis of variance (ANOVA) followed by Tukey’s *post-hoc* test was used to test the differences in the mass of dried digesta recovered after digestion, and in the FD4 and LY diffusion between each sample (bread or fermented product). Sample type (breads or fermented products), time (or stage of digestion) and the interaction between these two variables were used as fixed effects.

## Results

### Nutritional evaluation of the breads and fermented products

Table 1 shows the chemical composition of the raw materials (breads and fermented products, DM%). MB contained 12.9% of protein and WB 10.8%, whereas little protein remained after fermentation (0.4% for both FWB and FMB, on an as-fed basis). A difference of 3% in starch content was observed between the two bread types, with WB containing 74.9% of starch and MB 77.9%. MB contained more dietary fibre than WB, of which insoluble fibres were predominant (9.1 and 1.7%, respectively). For both breads, most of the dietary fibres were degraded during the fermentation process with 0.16 and 0.24% (on an as-fed basis) remaining for FWB and FMB, respectively.

**Table 1:** Nutrients and volatile fatty acids composition of the breads and fermented products studied.

	White bread		Wholemeal bread	
	WB	FWB	MB	FMB
Dry matter (%)	83.7	2.6	70.4	2.7
Ashes	2.4 *(2.0)*	4.6 *(0.1)*	2.8 *(2.0)*	4.1 *(0.1)*
Crude protein	10.8 *(9.0)*	15.3 *(0.4)*	12.9 *(9.1)*	15.0 *(0.4)*
Starch	74.9 *(62.7)*	–	77.9 *(54.8)*	–
Total dietary fibres	4.0 *(3.3)*	6.0 *(0.16)*	12.3 *(8.7)*	9.0 *(0.24)*
Insoluble dietary fibres	1.7 *(1.4)*	1.5 *(0.04)*	9.1 *(6.4)*	5.3 *(0.14)*
Soluble dietary fibres	2.3 *(1.9)*	4.5 *(0.12)*	3.2 *(2.3)*	3.7 *(0.10)*
Volatile fatty acids (mg/g DM)				
Lactic acid	11.0	41.0	5.5	29.8
Acetic acid	4.0	6.5	3.5	7.9
Propionic acid	–	2.5	–	1.2
Butyric acid	–	–	–	10.6
Total volatile fatty acids	15.5	50.0	9.0	50.0

**Notes.**

Abbreviations WB White bread FWB Fermented white bread MB Wholemeal bread FMB Fermented wholemeal bread

The sign “–” indicates that the nutrient or the volatile fatty acid was not detected.

Values for nutrients are presented as a percentage of dry matter and on an as fed-basis (in italic in brackets).

**Figure 1:**
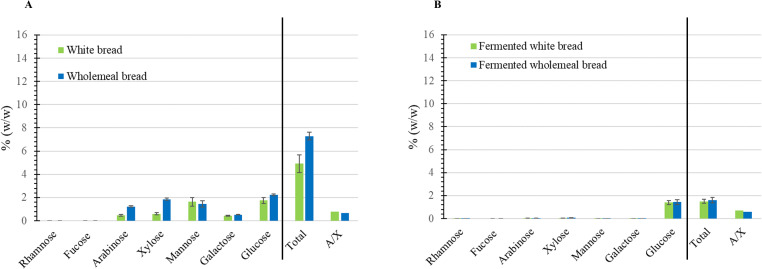
Neutral sugar composition of the bread before (A) and after (B) fermentation (% dry matter, w/w). Rha, rhamnose; Fuc, fucose; Ara, arabinose; Xyl, xylose, Man, mannose; Gal, galactose; Glc, glucose; Total, Total neutral sugars.

Neutral sugar analysis confirmed (Fig. 1) the higher dietary fibre content of the MB compared to WB, particularly for arabinose and xylose (arabinoxylan, the main cell wall constituent of wheat (Courtin & Delcour, 2002)). The ratio of these two sugars, that gives the degree of substitution of the xylan chain by arabinose residues, was rather high (0.79 for WB and 0.66 for MB), suggesting a high solubility and thus availability for bacterial fermentation. Fermentation led to the disappearance of most sugars from both breads, except of glucose (possibly from starch granules).

The volatile fatty acid profile of the two breads varied greatly in terms of composition (types of acid) and content. Before fermentation, both bread types contained lactic acid (11.0 mg/g DM for WB and 5.5 mg/g DM for MB) and acetic acid (4.0 mg/g DM for WB and 3.5 mg/g DM for MB). After fermentation, propionic acid was detected in both breads, but butyric acid was produced only in FMB (10.6 mg/g DM). The lactic acid content increased following fermentation to reach 41.0 mg/g DM for FWB and 29.8 mg/g DM for FMB. Overall, WB contained higher quantities of volatile fatty acids than MB.

### *In vitro* digestion and characterisation of the digesta

**Figure 2:**
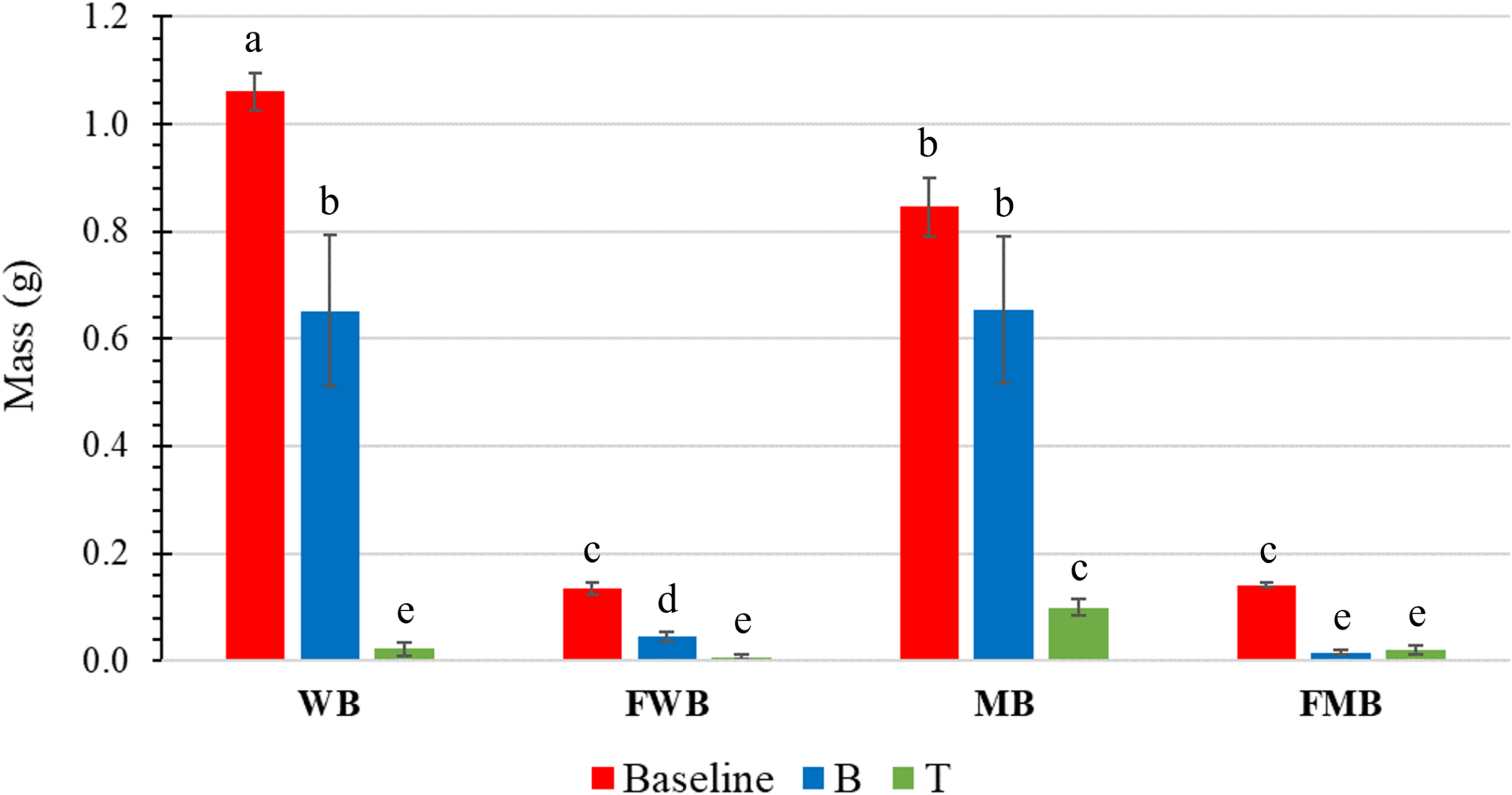
Dry mass of material at baseline (in red) and recovered after digestion without (B, in blue) and with (T, in green) enzymes (*n* = 9, 3 biological replicates measured 3 times). The differences between the samples were analysed using two-way ANOVA test followed by Tukey’s *post hoc*. Different letters indicate significant difference (*p* < 0.05).

Figure 2 presents the masses, reported in grams of dry matter, of the materials (breads and fermented products) recovered before and after oro-gastrointestinal digestion, without (B) or with (T) enzymes. In the absence of enzyme, there was an average loss in dry matter of 30% between the start and the end of digestion, however this difference was not significant for MB. The same observation was made for the fermented breads (samples B of FWB and FMB). When enzymes were present (samples T), a significant difference (*P* < 0.05) in the dry mass was observed between the baseline and after digestion (∼93% loss for both breads).

**Figure 3:**
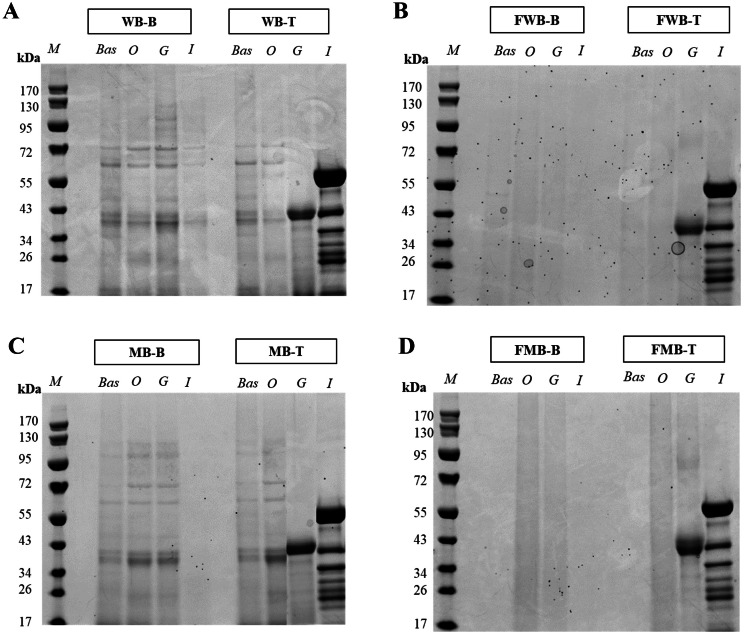
Images of the SDS-PAGE gels showing the protein and peptide profile of the breads (A and C) and fermented products (B and D) at baseline (Bas) and after each phase of digestion: oral (O), gastric (G), and intestinal (I). Samples B without enzymes and T with enzymes. M, Markers.

To gain more insight into the composition of these digesta, SDS-PAGE as well as volatile fatty acid and sugar analyses were performed SDS-PAGE analysis (Fig. 3) revealed distinct protein degradation patterns. At baseline, the protein profiles of WB and MB were slightly different. These proteins and peptides most likely come from the wheat flour, particularly the gluten, used to make the breads. On the other hand, the fermented breads contained few proteins which was in agreement with the mass recorded in Fig. 2. While a range of proteins and peptides of ∼70 and 40 kDa were found in the B samples for both breads, this was not the case in the presence of the enzymes (T). These proteins appeared to be fully hydrolysed in the gastric phase by pepsin activity (WB-T and MB-T samples G and I, in Figs. 3A and 3C). This complete protein hydrolysis contrasts with the partial degradation observed in enzyme-free conditions.

Table 2 summarises the volatile fatty acid and sugar composition of the digesta supernatants. The volatile fatty acid composition profile varied significantly (*P* < 0.05) according to the raw materials (breads or fermented products) and also to the type of digestion (with or without enzymes). Therefore, the total amount of volatile fatty acids was higher (*P* < 0.05) in the digestion occurring in presence of enzymes compared to the incubation without enzyme, and this for all samples except for FWB. Lactic acid remained relatively constant across conditions, while butyric acid increased, particularly in the wholemeal bread samples (MB-T and FMB-T).

**Table 2:** Composition in volatile fatty acids (VFA) and sugars of the supernatant of breads and fermented products after oro-gastrointestinal digestion, without (B) or with (T) enzymes.

	White bread				Wholemeal bread					
	WB-B	WB-T	FWB-B	FWB-T	MB-B	MB-T	FMB-B	FMB-T	SEM	*P*-value
VFA (g/L)										
Lactic acid	4.3	4	4.5	3.9	4	3.2	4	2.5	0.3	0.1
Acetic acid	0.5^a^	3.2^b^	0.5^a^	0.8^a^	–	–	–	2.6^b^	0.47	<0.05
Butyric acid	0.5^a^	4.5^b^	0.5^a^	–	–	12.8^c^	1.2^a^	8.2^c^	1.77	<0.05
**Total VFA (g/L)**	**5.3** ^**a**^	**11**.**7**^**b**^	**5.6** ^**a**^	**4**.**7**^**a**^	**4**.**0**^**a**^	**16.0** ^**b**^	**5.2** ^**a**^	**13**.**3**^**b**^	**1.78**	**<0.05**
Sugars (g/L)			
Maltose	9.7^b^	195.8^d^	–	70.7^c^	14.3^b^	170.4^d^	3.2^a^	68.4^c^	1.88	<0.05
Galactose	–	–	–	–	1.3^a^	8.1^b^	–	–	0.15	<0.05
Glucose	–	77.3^d^	–	18.0^c^	2.0^a^	66.9^d^	–	9.9^b^	0.85	<0.05
**Total sugars (g/L)**	**9.7** ^**b**^	**273.1** ^**d**^	**–**	**88**.**7**^**c**^	**17**.**5**^**b**^	**245.4** ^**d**^	**3.2** ^**a**^	**78**.**4**^**c**^	** 2.37**	**<0.05 **

**Notes.**

Abbreviations VFA volatile fatty acids WB White bread FWB Fermented white bread MB Wholemeal bread FMB Fermented wholemeal bread B Digestion without enzymes T Digestion with enzymes

The sign “–” indicates that the VFA or sugars were not detected.

a,b, c, d , e, f Total values for VFA and sugars are presented in bold. Values within a row with different superscripts differ significantly at *P* < 0.05.

Starch hydrolysis led to a greater increase in the sugars (maltose and glucose) produced in the T samples compared to the B samples, and this for both WB and MB. Despite a comparable quantity of initial starch between the two breads, the quantity of total sugars produced was greater for the white bread (*P* < 0.05), confirming that white bread is more digestible than wholemeal bread. The most abundant sugar among the three sugars measured in the digesta was maltose. The presence of this glucose dimer indicates that starch hydrolysis was incomplete. Indeed, since the enzymes of the intestinal brush barrier were lacking in our model, starch hydrolysis was not complete. The amount of sugars in the bread digesta was higher than in the digested fermented samples (*P* < 0.05). Some starch granules must have escaped fermentation as some maltose and glucose were produced after oro-gastrointestinal *in vitro* digestion.

### IPEC-J2 culture and viability after exposure with the digesta

The transepithelial electrical resistance was measured during the culture of the IPEC-J2; the values recorded increased until day 7 (up to around 3,000 Ω/cm^2^), corresponding to cell proliferation (see Fig. S2). From day 7 to 14, this resistance was stabilised when cells differentiated. The digesta produced in the *in vitro* digestion described above was then used as a substrate to test their impact on the IPEC-J2 viability. The MTS assay results (Table S1) demonstrated that detoxified digesta maintained high cell viability (>90% for most samples), with MB showing the greatest impact (80% viability). Based on these results and to ensure dietary fibre presence, the 1:10 dilution was selected for subsequent barrier function studies (as used in Perruchot et al., 2025; Grondin et al., 2025).

### Permeability evaluation of the IPEC-J2

**Figure 4:**
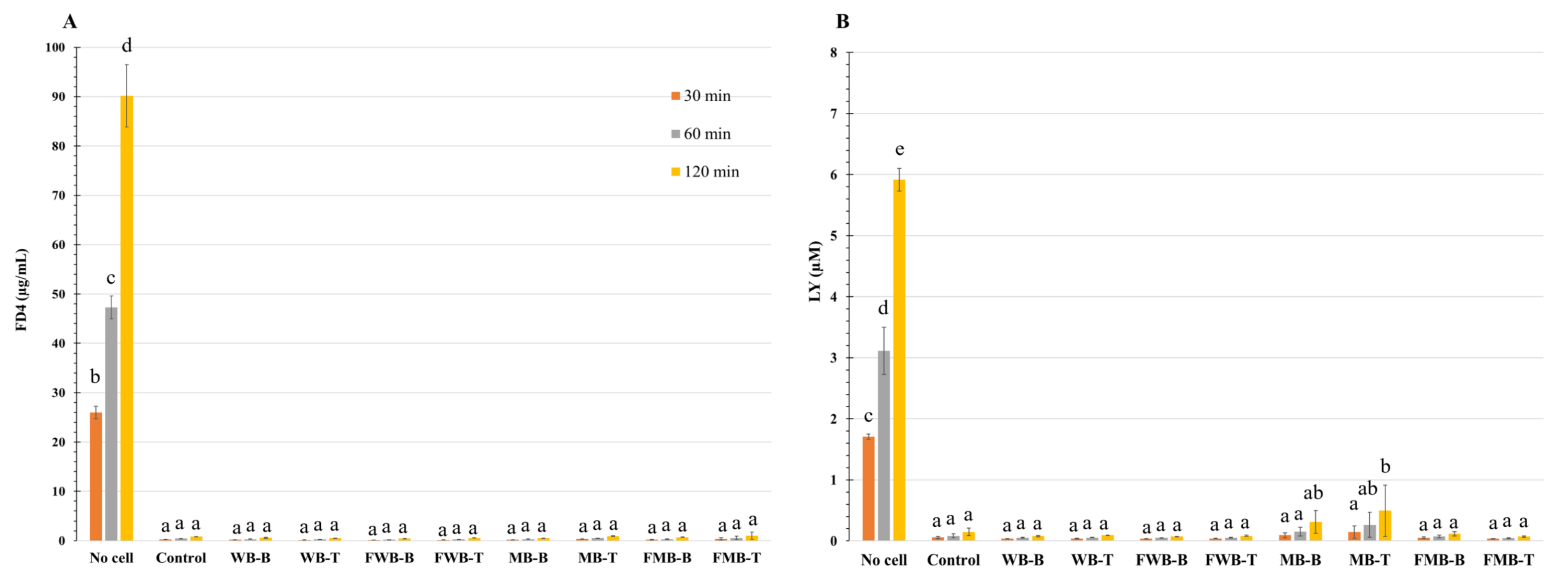
Overtime passage of FD4, in µg/mL (A), and LY, in µM (B) across the IPEC-J2 monolayers after incubation without cell, medium only (control) or the digesta of the breads or the fermented products (without, B, or with, T, enzymes). FD4 and LY concentrations are expressed as mean ± standard deviation (*n* = 3 biological replicates). The differences between the samples were analysed using two-way ANOVA test followed by Tukey’s *post hoc*. Different letters indicate significant difference (*P* < 0.05).

**Figure 5:**
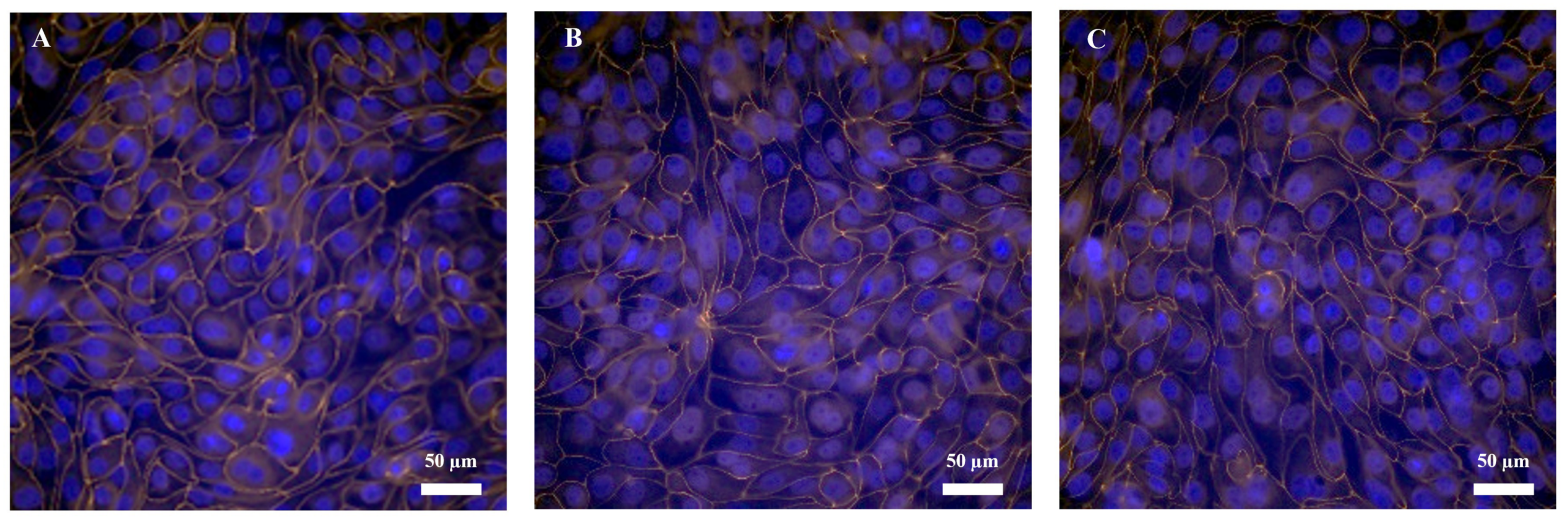
Representative images of ZO-1 immunostaining after 2 h of incubation with cell media only (control, A) or bread and fermented products digesta (white or wholemeal bread, (B) and fermented white or wholemeal bread, C) on IPEC-J2. Nucleus are stained in blue and the tight junction protein ZO-1 in red.

Permeability assessment using FD4 and LY revealed minimal barrier disruption for most treatments (Fig. 4). The passage of FD4 remained consistently low across all conditions after 2-hour exposure. However, LY permeability increased significantly (*P* < 0.05) in cells exposed to MB enzyme-present digesta (T sample), suggesting selective barrier modification. No other treatments showed significant differences from the control. Regarding ZO-1, immunostaining revealed no visually detectable differences in tight junction protein organisation between the digesta-treated cells and the control, suggesting that barrier modifications were functional rather than structural (Fig. 5).

## Discussion

This *in vitro* exploratory study investigated the impact of fermented bread on the barrier function in the jejunum of pig. Investigating the effect of fermentation products in the small intestine, particularly the jejunum, is of interest given that it is the main site of nutrient absorption (such as amino acids and small peptides). If intestinal health is maintained, such as intestinal barrier integrity and the absence of an inflammatory response, the intestinal permeability could be optimal thereby ensuring the absorption of hydrolysed nutrients by the enterocytes (Chelakkot, Ghim & Ryu, 2018).

This study addresses the knowledge gap regarding fermented bread’s potential as a sustainable feed additive (source of volatile fatty acids such as butyric acid known to have a positive impact on intestinal health (Feng et al., 2018)) for pig nutrition. Our primary objective was to evaluate the impact of fermented bread on intestinal barrier function using validated *in vitro* models (Grundy et al., 2022; Grondin et al., 2025). More specifically, our aims were (1) to perform a nutritional characterisation of the breads before and after fermentation in order to get an insight of the degradation of the breads following the fermentation process and the potential bioactive compounds produced, (2) to evaluation the degradation of the breads during *in vitro* oro-gastrointestinal digestion, and (3) to assess the effect of the *in vitro* digesta on porcine jejunal epithelial barrier function using the IPEC-J2.

### Fermented bread as a source of nutrients for pig

The breads were kept past their shelf-life for 7 days (a compromise for both white and wholemeal breads) before being fermented. The storage and fermentation conditions used in this work (liquid-phase at 35 °C for 8 days) were selected so as to be easily applied by farmers directly on their farms and to limit the environmental impact of the process.

As expected, the dietary fibre content of the breads decreased significantly after fermentation, suggesting that both insoluble (cellulose and certain hemicelluloses from the aleurone layer still present in the flour) and soluble (arabinoxylan and β-glucan) fibres were adequate substrates for the microorganisms present in our system (Saulnier, Guillon & Chateigner-Boutin, 2012; Fan et al., 2024). This fermentability was similar for the white and the wholemeal bread. The difference in volatile fatty acids obtained after fermentation between the two bread types is likely to be due to the initial fibre and starch composition (higher insoluble dietary fibre content for MB). The products of fermentation can be indicative of the types of microorganisms present (Betchem et al., 2024). Hence, the lactic acid content after fermentation of the two bread types could be explained by the action of lactic acid bacteria such as of the genus *Lactobacillus*, which transform sugars into lactic acid (Fan et al., 2024). The preferential production of butyric acid in wholemeal bread likely reflects its higher dietary fibre content, providing substrates for butyrate-producing microorganisms such as the anaerobe *Clostridium butyricum* (Dürre, 2014). The fermentation used in making the bread initiated the degradation of the wheat cell walls, having consequences on the microorganisms present (or more likely their spores) and the nutrients available for further fermentation (Guo et al., 2024). *Clostridium* can sporulate during bread baking and could be introduced by the bread itself. Lactic acid bacteria are ubiquitous microorganisms that can be found nearly everywhere, being sensitive to baking they are likely to have colonised the breads during storage (George et al., 2018).

Liquid-state fermentation was used in this work because it (i) facilitated the solubilisation of the dietary fibres and the degradation of the bread matrix, including the cell walls, compared to solid-state fermentation; (ii) limited contamination risks due to pH decrease caused by the production of volatile fatty acids; and (iii) enabled a better control of the fermentation process particularly the activity of *Clostridium butyricum* (Fan et al., 2024).

### *In vitro* digestibility of these potential new co-products

Studies performed in pigs showed that starch and protein digestibility is affected by the type of dietary fibres composing the feed (Ratanpaul et al., 2019; Zhang, De Vries & Gerrits, 2024). The particle size (proportion of intact or disrupted cell walls) of the flour also plays an important role in nutrient digestibility (Tagliasco et al., 2022). Even though not measured here, it could be expected that the wheat was ground to 180 µm as it is currently performed in the bread making industry. This is consistent with the low mass (*i.e.,* most of macronutrients hydrolysed) recovered after oro-gastrointestinal *in vitro* digestion of the two breads.

During staling, starch retrogradation takes place, which makes the starch resistant to upper gut digestive enzymes (Dymchenko, Geršl & Gregor, 2023). Given the low amount of residue recovered after WB hydrolysis, it is unlikely that any starch remained after digestion. In contrast, some macronutrients may have escaped hydrolysis for MB, probably due to an encapsulation mechanism by the intact cell walls (Grundy et al., 2022). Fermentation by degrading the cell walls can overcome this phenomenon, however the microorganisms will also utilise the macronutrients leaving little nutrients for the animal (Fan et al., 2024). Moreover, fermentation reduces other anti-nutritional factors such as tannins, phytate and trypsin inhibitor, contained in the feed (Zentek & Goodarzi Boroojeni, 2020). In grow-finisher pigs, cereal fermentation has been shown to improve either the ileal digestibility or the total tract digestibility of dry matter, organic matter, and crude protein (Jørgensen et al., 2010; Torres-Pitarch et al., 2020).

The low pH of the fermented products (pH 3) can facilitate pepsinogen conversion to pepsin, thereby promoting protein hydrolysis in the gastric compartment (Pluske, Turpin & Kim, 2018; Nowak et al., 2021). Both butyric and lactic acid present in the fermented breads therefore play important roles in the digestive function of piglets. Changes in butyric acid content following digestion likely reflect shifts in dominant microorganisms due to pH fluctuations between digestive phases (oral, gastric, and intestinal) and temperature variations (from 37 °C for the fermentation to 39 °C during digestion).

Overall, our data revealed that our fermentation method resulted in an important degradation of the bread matrix, with most of the nutrients being used by the microorganisms, which led to highly digestible co-products. Some starches must have resisted fermentation, as indicated by the maltose produced during FMB digestion, but no peptides were visible in the SDS-PAGE gels. These fermented products are thus a source of volatile fatty acids and dietary fibres (including resistant starch), but not of protein. Caution ought to be taken when comparing the digestibility between the breads and the fermented products as the breads were solid at the beginning of the *in vitro* digestion whereas the fermented products were liquid (with particles of undegraded breads). Given the high differences in composition and texture between those two types of products using the same weight (five g and five mL) was the compromise that had to be made.

### Impact of the fermentation on the barrier function of the IPEC-J2

Limited literature exists regarding fermented products’ effects on intestinal health in the upper gastrointestinal tract (Alberge et al., 2024; Wang et al., 2025). Most studies investigating the effect of fermented cereals on intestinal health explore their action in the colon *via* the microbiota (Torres-Pitarch et al., 2020). However, a compromised barrier function in the duodenum and jejunum will hinder nutrients uptake, which in turn can affect the animal’s growth and overall health (Pluske, Turpin & Kim, 2018). Supplementation of pig diets with fermented wheat bran has been shown to improve intestinal health: enhanced intestinal integrity in the duodenum and diversity of the colonic microbiota compared to the control diet (He et al., 2023).

In the colon, the role of butyrate is well-documented in humans, rats and pigs, where volatile fatty acids are produced through the microbiota fermentation (Feng et al., 2018; Van der Hee & Wells, 2021). Butyric acid can then be recognised by specific receptors (GPR43 and GPR41) and serve as an energy source for intestinal cells. Butyric acid can also induce an anti-inflammatory response *via* stimulation of interleukin-10 production. Its mode of action against inflammation through mediation of signalling pathways involving nuclear NF-κB and inhibition of histone deacetylase was also previously reported (Bach Knudsen et al., 2018). Moreover, butyric acid can reinforce the integrity of the intestinal barrier *via* the upregulation of tight junction proteins (Feng et al., 2018; Connolly, Sweeney & O’Doherty, 2025; Wang et al., 2025). To achieve this latter effect in the small intestine, where the microbiota is less abundant and the dietary fibres remain intact, butyric acid can be provided directly in the feed.

Our results showed that for MB the diffusion was partial, where only small molecules (LY of 522 Da, equivalent to the size of a peptide of 4–5 amino acids) diffused through the IPEC-J2 monolayer. The paracellular space must have been affected by this sample but not sufficiently to allow larger molecules to diffuse, also the tight junction proteins did not seem to have been compromised. This effect disappeared once the bread was fermented suggesting that either the insoluble dietary fibre fraction of MB could have reduced cell adhesion (an “abrasive” effect) or the butyric acid in FMB may have strengthened the intestinal epithelial barrier (Bach Knudsen, Hedemann & Lærke, 2012; Kovanda et al., 2025).

Incorporating fermented bread into piglet diets as a natural source of bioactive compounds, particularly butyric acid, represents a promising alternative to synthetic molecules and may contribute to reducing antibiotic use (Saettone et al., 2020). Further studies should comprehensively evaluate the potential toxicity of fermented products before feeding them to pigs which should include microbial characterisation. While the presence of lactic and butyric acids suggests dominance by *Lactobacillus* and *Clostridium butyricum*, respectively, this requires microbiological confirmation. Mold can develop during bread storage particularly for wholemeal bread, however, the fermentation appeared to reduce this phenomenon possibly by destroying the pathogens. Molds are more likely to occur in solid-state fermentation (Zentek & Goodarzi Boroojeni, 2020; Sandoval et al., 2024). Again, further analyses are required to confirm this for our fermentation conditions.

## Conclusions

This study presents for the first time an *in vitro* investigation of the effect of fermented bread on jejunal barrier function. The combination of oro-gastrointestinal digestion simulation and IPEC-J2 cell culture successfully confirmed our hypothesis that fermented breads have the potential to address physiological challenges occurring in piglets, notably intestinal disorders after weaning. The *in vitro* models employed in this work enabled us to understand the mechanisms underlying digestibility and intestinal health impacts of novel ingredients while contributing to the reduction of animal experimentation, supporting the principles of replacement, reduction, and refinement in research.

##  Supplemental Information

10.7717/peerj.21397/supp-1 Supplemental Information 1 Raw data.

10.7717/peerj.21397/supp-2 Supplemental Information 2 Supplementary Figures and Tables.

## Additional Information and Declarations

### Data Availability

The following information was supplied regarding data availability: The raw data is available in the Supplemental File.
