# Supplementary material for: Fermented bread, a promising additive to pig feed to improve intestinal health—an in vitro study
Source: PeerJ. 2026 Jun 9;14:e21397. doi: 10.7717/peerj.21397 (PMC13262544; doi:10.7717/peerj.21397)
Supplement: Supplemental Information 2 [file peerj-14-21397-s002.docx]

**Supplementary material**

**Table S1 IPEC-J2 viability after 2 h of exposure with the control (HBSS) and the different digesta**

| Cell viability (digesta % vs control) | | |
| --- | --- | --- |
| Control (cells in HBSS) | 100 ± 9.1 | |
| Dilution (v/v) | B | T |
| WB |  | |
| 01:10 | 104.0 ± 6.7 | 93.6 ± 6.0 |
| 01:20 | 102.2 ± 4.3 | 96.4 ± 5.6 |
| FWB |  | |
| 01:10 | 87.1 ± 4.8 | 89.9 ± 5.3 |
| 01:20 | 93.6 ± 7.3 | 83.5 ± 3.6 |
| MB |  | |
| 01:10 | 106.2 ± 6.2 | 79.8 ± 5.1 |
| 01:20 | 86.8 ± 6.5 | 80.8 ± 4.8 |
| FMB |  | |
| 01:10 | 88.4 ± 10.4 | 82.1 ± 3.2 |
| 01:20 | 83.9 ± 3.9 | 86.7 ± 5.1 |

**
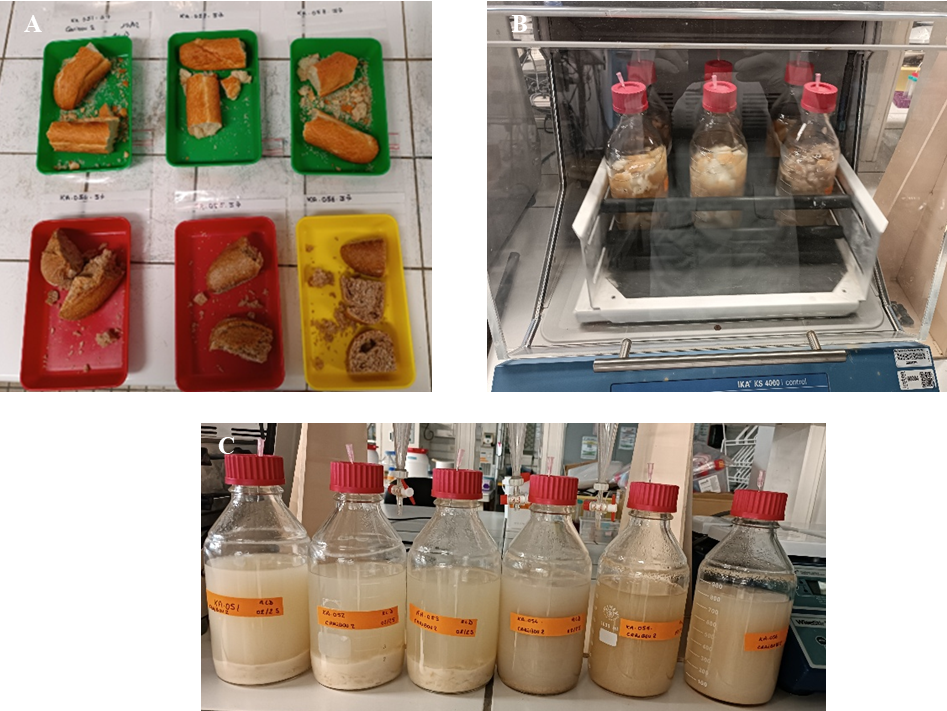
**

**Fig. S1**. Bread used in this study at baseline (A), during (B) and after (C) fermentation

**Fig. S2**. Evolution of the transepithelial electrical resistance during cell growth and differentiation
